# Are government incentives effective for avoided deforestation in the tropical Andean forest?

**DOI:** 10.1371/journal.pone.0203545

**Published:** 2018-09-13

**Authors:** Pablo Cuenca, Juan Robalino, Rodrigo Arriagada, Cristian Echeverría

**Affiliations:** 1 Grupo de Investigación Bosques Tropicales y Cambio Global, Universidad Regional Amazónica Ikiam, Tena, Ecuador; 2 Laboratorio de Cambio Global, Universidad Regional Amazónica Ikiam, Tena, Ecuador; 3 School of Economics, Universidad de Costa Rica, San José, Costa Rica; 4 Centro Agronómico Tropical de Investigación y Enseñanza (CATIE), Turrialba, Costa Rica; 5 Department of Ecosystems and Environment, Pontificia Universidad Católica de Chile, Santiago, Chile; 6 Center for Applied Ecology and Sustainability (CAPES), Santiago, Chile; 7 Millennium Nucleus Center for the Socioeconomic Impact of Environmental Policies (CESIEP), Santiago, Chile; 8 Laboratorio de Ecología de Paisaje, Facultad de Ciencias Forestales, Universidad de Concepción, Concepción, Chile; University of Vermont, UNITED STATES

## Abstract

In order to ensure the provision of goods and services from forests, many governments have promoted less-traditional conservation initiatives such as programs of payments for ecosystem services called, more broadly, direct payments for conservation. The Socio Bosque Program (SBP) is a governmental program in Ecuador that directly provides economic incentives to rural families and local and indigenous communities who have voluntarily agreed to comply with some conservation activities. An impact evaluation method (matching) was used to assess the impact of the SBP between 2008 and 2014. This study revealed that on average, the SBP reduced deforestation by 1.5% in those forests that received the SBP’s direct payment. These forests would have been deforested if the SBP had not been implemented. Assessment of the impact of the SBP on individual and collective contracts, using the matching method, revealed that 3.4% and roughly 1% of the forest would have been deforested in the absence of the program, respectively. In other words, the protected area in the collective SBP was 1,247,500 ha and, if the SBP had not been implemented, an area of 11,227 ha would have been lost between 2008 and 2014. The 165,700 ha protected by the individual SBP, it was estimated that 5,733 ha were not deforested due to the implementation of the conservation program. Conventional estimates of the impact of the SBP tend to overestimate avoided deforestation because they do not control for observable covariates that correlate with or affect both SBP participation and deforestation. The conclusions are robust, even given potential hidden biases. The present study demonstrated that the SBP serves to mitigate the effects of climate change, especially with those contracts that are intended for individual owners.

## Introduction

Tropical forests play an important role in the regulation of the global climate system through the storage of large carbon reserves [1, 2], and the regulation of energy and water flows [3]. The release of carbon into the atmosphere due to deforestation and forest degradation is the second-largest source of greenhouse gas (GHG) emissions [4]. The XIII Conference of the Parties to the United Nations Framework Convention on Climate Change created the Bali Action Plan, which emphasized the need to search for incentive mechanisms for developing countries to reduce carbon emissions from deforestation and forest degradation (REDD) [5].

Governments play an important role in the mitigation of GHGs, because forests are keystones in the provision and regulation of ecosystem services such as carbon storage [6, 7]. Accordingly, to ensure the provision of forest ecosystem services, some governments have implemented mechanisms such as payments for ecosystem services; these have been widely documented [8–13] and are considered the most direct way to comply with conservation goals [14].

Conservation professionals and decision makers have begun to more carefully assess the impact of payments for ecosystem services, funded by national governments, on social and environmental outcomes [15–17], and how these impacts vary spatially [18]. Despite these efforts, little is known about the impact of payments for ecosystem services [16].

One of the challenges of assessing the impact of payments for ecosystem services relates to determining the rate of deforestation that would have occurred had the program not been implemented, called the counterfactual scenario [19], which is known as the casual effect of the program when compared with the actual rate of deforestation that occurs with the program [20, 21]. Clearly, it is impossible to observe the two outcomes in the same individual participant at the same time. If the program did not exist, the outcome of the individual participant (i.e., the counterfactual scenario) would be hypothetical and, consequently, not directly observed [22, 23].

Most assessments of the impact of payments for ecosystem services and conservation policies have, in general, been conducted mainly in Costa Rica, Mexico, and Indonesia [9, 18, 19, 24–27], and very few studies have been conducted in the tropical Andean forest [28–30], which is recognized for its high capacity to mitigate the effects of GHGs and its huge biological diversity. A robust assessment of the impact of payments for ecosystem services on these ecosystems will make it possible to improve the design and focus of conservation programs, thus avoiding the release of carbon into the atmosphere due to deforestation and degradation of the tropical Andean forest.

We chose Ecuador because it is widely known for being the most mega-diverse country per unit of area [31, 32]. Currently Ecuador is a leader in the debate to have ‘avoided deforestation credits’ recognized by international climate-change conventions. It also had one of the top deforestation rates during the 1980s and 1990s [33], driven mainly by the expansion of cattle grazing, agrarian reform and cacao and banana production [34, 35]. In 1980, Ecuador had approximately 15.5 million hectares of forests, and from 1990 to 2014, continental Ecuador lost approximately 1.8 million hectares of forest and approximately 5.8 million hectares were assigned to legal protection [36].

The Ecuadorian SBP program is an example of a conservation program aimed at preventing the loss of forests and maintaining carbon reserves [37]. The SBP is likely to enable storage of 722 million tons of CO2eq [38] in the whole of Ecuador. Currently, Ecuadorian carbon reserves are approximately 1,500 million tons, and the equivalent carbon dioxide not emitted into the atmosphere is 5,621 million tons [39]. Thus, the goal of the SBP is to protect around 3.6 million ha of native forest and other ecosystems of global importance for biodiversity conservation, thereby reducing rates of deforestation and associated GHG emission and increasing incomes and protecting human capital in communities with high poverty rates [6, 37].

To date, 55.6 billion dollars have been invested in SBP, and 1.5 million ha of forests, moors and mangroves have been protected [37]. Landowner contracts are for 20 years, and incentives are based on the amount of land enrolled, not its value. For example, the first 50 ha of land receives US$30 ha^-1^ year^-1^, the second 50 ha receives US$20 ha^-1^ year^-1^ and so on [40, 41]. Even though the goals of the SBP are targeted at nature and society [42], its impact on the deforestation of the tropical Andean forest is unknown, although these ecosystems are recognized for their high capacity to mitigate GHG effects and their biological diversity [31, 32, 43]. Socio Bosque is part of Ecuador’s national REDD+ strategy, which is currently under construction, specifically under the component of incentive-based policies, in this case for the conservation of forests. The SBP furthermore sheds light on possible benefit-sharing mechanisms for REDD+ [6].

The SBP has great potential to reduce deforestation and ensure carbon stocks by the conservation of tropical Andean forest, and therefore we estimated, for the first time, the impact of this program via individual and collective contracts on loss of forest area. The SBP was selected because it is a government-funded national program that transfers economic incentives directly to rural families and to local and indigenous communities that have voluntarily agreed to comply with clearly agreed-upon conservation activities [6].

More specifically, the goal of the present study was to estimate the impact of the SBP on avoided deforestation in the tropical Andean forest between 2008 and 2014. The measurement of the impact of the SBP was based on the estimation of a counterfactual scenario, which allowed us to answer the following research question: How much tropical Andean forest would have been lost in the absence of the SBP? The results of the present study showed that SBP was more effective for individual contracts than it was for collective contracts. This information will allow decision makers to improve the allocation of financial resources, as well as to adapt and focus the efforts of the SBP within the national framework of financial sustainability, biodiversity conservation and climate change.

## Materials and methods

### Data

**Fig 1:**
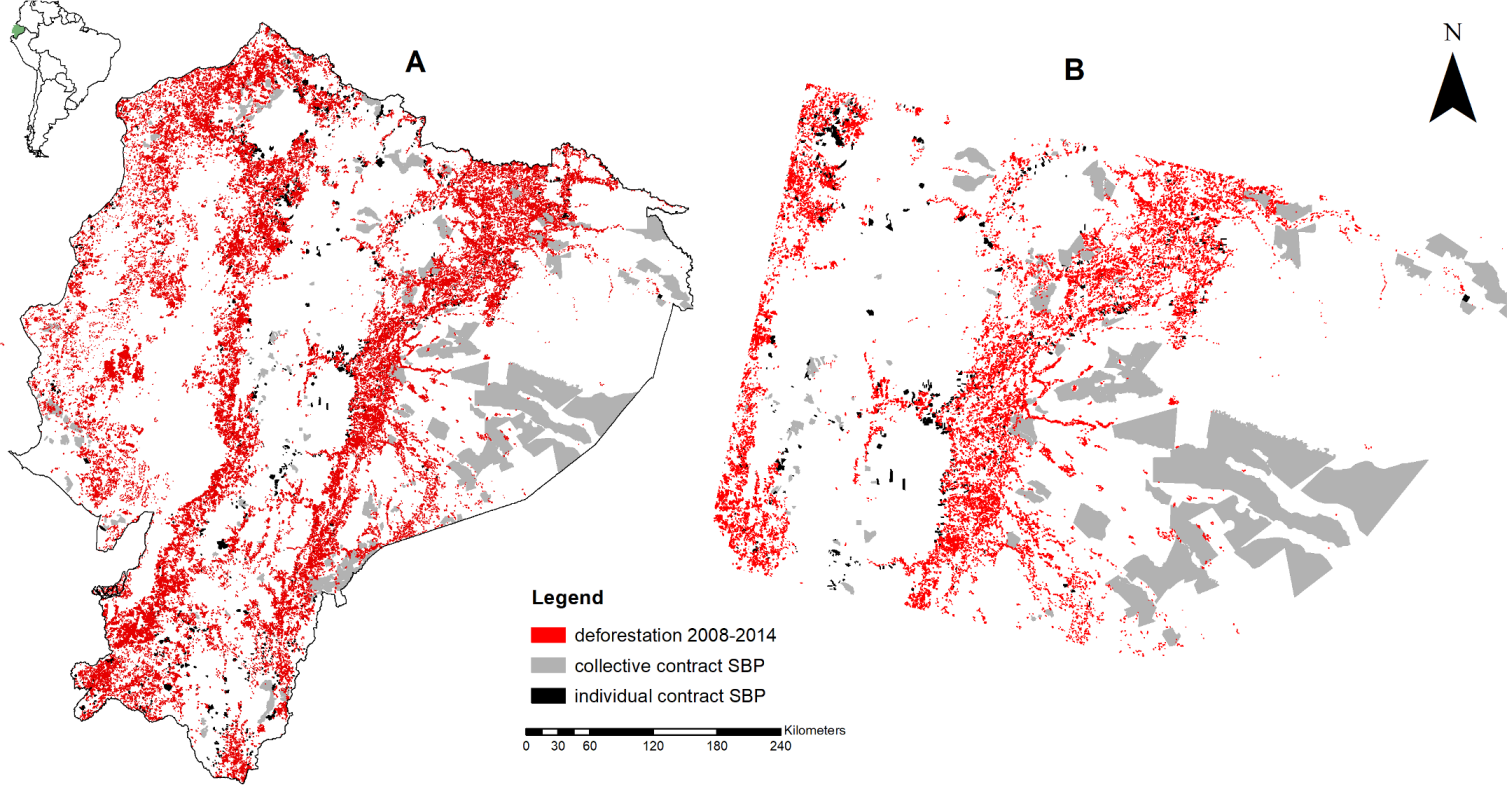
Locations of the study area (A). Individual contracts are located closer to deforested area than collective contracts (B).

The present study estimated the causal impact of the SBP on avoided deforestation after 2008 at the national level in continental Ecuador (Fig 1). To that end, we used a random sample of 300,000 units of observation of 30 x 30 m on the map of Ecuador. The final sample of the analysis included 279,000 cells, because we excluded cells that were not forests in 2008. In addition, the random sample was focused on two types of SBP contract (i.e. individual and collective).

The dependent variable was deforestation between 2008 and 2014. Any area with forest in 2008 that was converted to non-forest in 2014 was considered deforested and was assigned a value of 1. Areas covered by forest in 2008 that were not converted to non-forest in 2014 were assigned a value of 0.

To estimate the change in forest cover, we used maps based on Landsat-5 TM satellite images obtained from 2008 to 2014. These maps were generated by the SBP and contrasted *in locus* during 61 field trips, during which 699 reference points, 3,591 calibration points, and 1,245 validation points were recorded [36, 37].

With respect to SBP contracts (the SBP provides payments per hectare to forest owners biannually based on 20-year contracts), we received spatial information from the Ecuadorian Ministry of the Environment (MAE) that included all members who were involved in the program for each year during the period of analysis (2008–2014), including individual and collective SBP contracts.

To properly characterize the treated groups and to find similar untreated groups, the information about land cover was combined with spatially explicit information on covariates that affected both the areas with and without the SBP.

In this way, we resolved the key problem faced by observational studies (i.e., the confounding factors that might influence the estimation of the effects of treatment) [44]. Based on the literature review regarding the effectiveness of conservation policies [19, 28, 45–47], we used the following confounding variables: altitude, slope, distance to national roads, distance to local roads, distance to villages, distance to rivers, distance to forest borders, average rainfall and average temperature. These variables were generated from information maps of the MAE.

### Methods

Propensity Score Matching (PSM) was implemented using one-to-one, one-to-two and one-to-three nearest neighbor matches, without replacement [19, 48] and by using a proper control group to estimate what would have happened if the treatment―in this case, the SBP―had not been implemented [22, 49]. The PSM was defined as the probability that one cell might be allocated for conservation under SBP. To estimate this probability, we assigned the value 0 to cells without the SBP and 1 for cells with the SBP. The matching technique [50–52] was used to match cells with the SBP (or “treatment cells”) with cells without the SBP (also called “control cells”) that had the most similar probability.

The PSM allowed reduction of the differences between the treated group and the control group in the set of characteristics that influence the propensity to be in a cell with the SBP, which, in turn, affect the variable of impact of interest (deforestation, in this case).

To improve the covariance balance, we complement the propensity score matching using another matching algorithm called “matching with calipers” which consisted of defining a level of tolerance to judge the quality of the matching. [50]. This alternative matching algorithm allowed us to verify the robustness of the results to different matching strategies. Thus, if a treated cell matched an untreated observation whose propensity score was not within the caliper, it was excluded from the analysis.

Following Pfaff, Robalino [53], we adjusted for remaining bias by running OLS with the matched sample. This controls for remaining sources of bias after matching.

### Test of differences in standardized means

To judge if the matching technique improved the similarity between the observations of the treatment and control groups, we performed a balance test of covariates in the sample before and after the matching procedure through calculation of normalized differences. This is preferable to *t* statistics when there are large differences in sample size [54]. It is specifically calculated as follows:

X¯t−X¯cσt2+σc2

where $\bar{X}$ is the mean, *σ*_*t*_^2^ is the variance within the SBP, *σ*_*c*_^2^ is the variance outside the SBP, “t” means land within the SBP (treatment) and “c” land outside the SBP (control). The golden rule of difference in normalized differences is that, when it is greater than 0.25, it may distort the regression estimate [55].

### Sensitivity test

When the PSM is used, the main identifying assumption is that there are no non-observed variables that affect the probability of conservation and, at the same time, deforestation. In the case that such non-observable variables occur, the differences observed between cells with and without the SBP cannot be entirely attributed to the program, and the estimate of impact will be biased.

In order to test the robustness of the impact estimates, we used the sensitivity analysis proposed by Rosenbaum [49] and Wilcoxon’s test, which assumes that each unit of observation has a fixed value for a non-observed covariate (or a composite of non-observed covariates). The higher the range level (Γ) in which the inference of the estimated effect of the SBP on deforestation does not change, the more confidence in the conclusion that the estimation of the causal effect is not affected by a non-observed difference [55].

## Results

Table 1 shows the impact estimates of the SBP on avoided deforestation. The measurement of avoided deforestation using a conventional method (without controlling for other variables) revealed that 5.1% of the cells with the SBP would have been deforested between 2000 and 2014 had they not been included in the SBP in 2000. The estimate of avoided deforestation using the PSM technique revealed that between 1.4% and 1.5% of the cells with the SBP would have been deforested between 2000 and 2014 in the absence of the program. When we used matching with calipers, the estimate of the impact of the SBP on avoided deforestation was 1.4% (*p* < 0.01). The analysis using one-to-one, one-to-two and one-to-three nearest neighbor matching without replacement of the impact of the SBP indicated that it does vary widely for individual and collective contracts. The results show that 3.4% and roughly 1% of the forest would have been deforested in the absence of the SBP for individual and collective contracts, respectively.

**Table 1:** Estimated impacts of SBP on avoided deforestation.

	SBP	SBP-collective	SBP-individual
No. of program cells (treatment)	30,439	27,733	2,706
No. of control cells	260,050	260,050	260,050
***Conventional Approach***			
Naive *t*-test	-0.0511^a^	-0.0525^a^	-0.056^a^
OLS with covariates	-0.0219^a^	-0.0182^a^	-0.0568^a^
***Matched sample using Propensity Score Matching (PSM)***^***b***^			
PSM (n = 1)	-0.0150^a^	-0.0090^a^	-0.0346^a^
PSM (n = 2)	-0.0143^a^	-0.0084^a^	-0.0328^a^
PSM (n = 3)	-0.0146^a^	-0.0085^a^	-0.0334^a^
Calipers (c = 0.01) (n = 1)	-0.0143^a^	-0.0090^a^	-0.060^a^
***Bias adjustment using PSM matched sample***			
PSM (n = 1)	-0.0154^a^	-0.010^a^	-0.0801^a^
PSM (n = 2)	-0.0137^a^	-0.0093^a^	-0.0787^a^
PSM (n = 3)	-0.0140^a^	-0.094^a^	-0.0799^a^

Note.

^a^Significant estimates—*p* < 0.05

^b^Standard error for post-matching estimates using the variance formula (Abadie & Imbens, 2008).

Given that differences within some covariates might still remain after matching, we also ran a regression using the matched PSM sample to eliminate any remaining bias (see Table 1 bias adjustment using PSM sample). The results for all SBP contracts and for the SBP-collective contracts do not vary substantially. However, the estimated impact of SBP individual contracts more than double reaching 8 percentage points. These results confirm our hypotheses that SBP individual contracts have a significant and negative effect on deforestation and that these effects are significantly larger than SBP collective contracts.

If the areas with and without the SBP differ in characteristics that not only affect the conservation of the forests but also determine changes in forest cover, these differences could not be entirely attributed to the program [16]. The comparisons of the differences in the distribution of the covariates between cells with and without SBP showed significant differences in the means before matching. Therefore, if the results of the matching technique were effective, these two measures (fifth column in Table 2) should be zero or close to this value in the matched sample. Analysis of the covariates using the matching technique indicated that the balance of covariates improved substantially after matching, and biases in observable variables were minimized (Table 2).

**Table 2:** Balance of covariates.

Variables	Sample	Mean value with SBP	Mean value without SBP	Differences in mean values	Raw eQQ difference	Normalized differences
Slope (°)	Unmatched	10.023	12.41	-21.94	2.3905	-0.1496
	Matched	10.023	9.949	0.6803	0.1350	0.0236
Altitude (masl)	Unmatched	560.45	802.29	-39.775	257.49	-0.2644
	Matched	560.45	552.45	1.3155	9.5068	0.0061
Distance to national roads (km)	Unmatched	81.616	41.083	65.638	40.525	0.5528
	Matched	81.616	80.684	1.509	2.0586	0.0295
Distance to local roads (km)	Unmatched	48.564	23.217	68.138	25.342	0.5947
	Matched	48.564	47.855	1.9059	0.7863	0.0554
Distance to villages (km)	Unmatched	12.278	9.2756	39.981	3.0604	0.2794
	Matched	12.278	12.175	1.3684	0.1681	0.0167
Distance to rivers (km)	Unmatched	7.9365	8.0214	-1.2433	1.7523	-0.0101
	Matched	7.9365	7.1594	11.38	0.7921	-0.0100
Distance to forest borders (km)	Unmatched	6.1542	3.4012	55.436	2.7623	0.4146
	Matched	6.1542	5.2885	17.433	0.8181	0.0104
Average rainfall (mm)	Unmatched	2789	2610.7	25.497	282.1	0.1502
	Matched	2789.7	2786.6	0.4448	10.916	0.0115
Average temperature (°C)	Unmatched	23.277	22.204	37.863	1.5169	0.1446
	Matched	23.277	23.299	-0.7518	0.0757	-0.0026

Matching decreased differences in the distribution of all covariates to a large extent, as shown by the differences in mean values and normalized differences in Table 2 [56].

The results of Table 1 are also robust to different matching strategies. In particular, when using different numbers of matched control units by treated observation (the closest neighbor, one-to-two or one-to-three), the results do not vary significantly.

To put these findings into context, the protected area in the collective SBP was 1,247,500 ha and, if the SBP had not been implemented, an area of 11,227 ha would have been lost in 2014. On the other hand, of the 165,700 ha protected by the individual SBP, it was estimated that 5,733 ha were prevented from being deforested due to the implementation of the conservation program.

### Sensitivity test

The non-observed heterogeneity between the conserved and non-conserved areas by the SBP is presented in S1 Table. The range changes the probability of rejecting the hypothesis. The higher the range level (Γ) in which the inference of the estimated effect of the SBP on deforestation does not change, the more confidence in the conclusion that the estimate of the causal effect is not affected by a non-observed difference (S1 Table). In other words, the second column in Annex C indicates that the estimate that 1.5% of deforestation was deterred by the SBP remained significant and different from zero, even in the presence of moderate non-observed biases.

## Discussion

The present study estimated the amount of avoided deforestation resulting from the payment policy for conservation called the SBP. In order to avoid bias estimates of program impact due to the lack of randomness in the treatment assignment (i.g. participation in SBP) and to obtain an unbiased estimate of the effect of this conservation policy, we used the matching method, as has been done in other empirical studies [15, 28, 45, 57]. According to Robalino, Sandoval [19] and Blackman, Pfaff [58], the matching method allows for control of the selection bias in estimates of avoided deforestation, because the allocation of conservation policies (e.g., SBP, protected areas, payment for environmental services) is not performed randomly.

Based on the matching technique, we can answer the counterfactual question regarding what would have happened in the tropical Andean forest had the SBP not been implemented. The study revealed that the SBP was effective at preventing the loss of forest areas. The program was more effective in forests with individual contracts than in forests with collective contracts. If the SBP had not been implemented, between 1.5% and 3.4% of forests would have been deforested in 2014. These results are consistent with the percentages of avoided deforestation reported by other studies that assessed the impact of similar conservation policies [8, 15, 25, 28, 58].

Although the present study did not focus directly on the difference in SBP impacts between individual and collective contracts, we assume that this gap can be explained by linking deforestation pressure (deforestation rate in the absence of protection) and the observed deforestation rate under the SBP [58]. According to MAE [38], the majority of the land covered by individual contracts is located 500 meters from a deforested area, and deforestation pressure is higher than for land covered by collective contracts. Under these circumstances, the individual SBP contracts are more effective at helping to avoid deforestation when compared to the counterfactual assumptions of what would be expected in their absence. Conversely, collective contracts reduce deforestation in areas with relatively low pressure of forest loss. The literature on common property and collective-community management of forests has generally been positive with respect to the potential for safe-land ownership to support customary standards and local institutions [59]. The literature is full of examples of institutions/associations for community management that have been successful in land management [6, 59, 60]. The majority of the examples cited provide a rich descriptive inference about the design and operation of the institutions at the community level; however, they do not necessarily reveal explicit counterfactual outcomes that allow the impact of conservation programs to be estimated. The present study contributes to the literature that assesses conservation programs based on robust methods and reveals that, at the individual level, the SBP is quite effective [17].

When the effectiveness of conservation policies is estimated, studies may try to make comparisons between conservation policies implemented within and outside the relevant areas, or to compare the outcomes obtained before and after the establishment of the conservation policy [61–63]. According to Andam, Ferraro [45], these estimates may be strongly biased. To illustrate this fact, in the present study, conventional methods would have estimated the avoided deforestation due to the SBP to be between 5% and 7% (i.e., two to three times higher impact of the SBP than that reported by the matching technique). Estimates of the impact of the SBP on deforestation were significantly lower than those generated by conventional methods, which do not control for the lack of randomness in the treatment assignment [8, 53]. According to Blackman, Pfaff [58], these estimates are a common finding in the assessment of forest conservation policies that use the matching technique and other quasi-experimental strategies [19, 57, 58, 64, 65].

As expected, authors of previous studies were not aware of the use of the matching technique to address the factors that might hide the relationship between SBP and avoided deforestation. This fact has characterized multifold investigations on SBP due to a weak empirical basis [66, 67].

Since the creation of the SBP, the Ecuadorian government has invested around 56 million dollars in collective and private contracts to achieve its conservation goals [68]. The present study suggests that this investment should be increased if a reduction in the deforestation rate is one of the primary goals of the SBP, given that substantial rates of forest loss have been reported in Ecuador [28, 30, 69]. On the other hand, the positive impact of the SBP on deforestation at the local level is an indicator of Ecuadorian efforts to preserve the tropical Andean forest according to its new Constitution, which established that the State will reduce deforestation and preserve the forests (Article 414) [70]. At the global level, the results indicate that the conservation efforts being made by Ecuador to avoid deforestation are in line with the efforts of various other countries [15, 45, 55, 58, 71, 72].

According to Cao, Zhong [42], mechanisms that consider the contribution of both environmental and human aspects to human development are more likely to provide appropriate long-term measures through which people can escape from poverty than those that consider human aspects alone. Such mechanisms can improve both nature and society. It seems that the SBP could be such a mechanism [6].

### Implications of the study

The role played by tropical forests in the climate and carbon cycle has a direct relationship with current/future deforestation rates and the amount of forest that remains preserved or could increase carbon reserves [73]. This reveals the importance of measuring avoided deforestation in a robust way to prevent under or overestimates of policy impact. For example, an overestimate of avoided deforestation might indicate that the goals of the SBP have been fulfilled; however, this may not be the case. In contrast, there might be higher deforestation rates than those reported by conventional methods.

On the other hand, the SBP is part of the REDD+ national strategy, which is being implemented in Ecuador in the form of credits from avoided deforestation in order to preserve forests [74, 75]. In such schemes, estimating avoided deforestation in a robust way is of paramount importance, to allow buyers or sellers to negotiate credits from GHG emissions. This procedure should be performed through correct calculations and without diverting efforts to maintain the carbon resulting from forest conservation [45].

The assessments of impacts―such as those performed in the present study―could use behavioral experiments and field surveys (aspects not addressed in the present study) to connect incentives, institutions and decisions on the use of the soil with the outcomes of avoided deforestation in the tropical Andean forest [64].

Based on the findings of the present study, the SBP could improve its design by: 1) studying the optimal distance at which to achieve efficient interaction among conservation policies, 2) analyzing the relationship between deforestation pressure and SBP location (i.e. what would have happened had the SBP not been implemented), 3) identifying sensitive SBP sites at which higher rates and pressures of deforestation were observed (i.e., where the SBP claims greater avoided deforestation) and 4) incorporating a landscape approach in which the SBP payment of the SBP allows the integration of landscape conservation with sustainable development.

## Conclusion

The SBP is an initiative to preserve forests and reduce GHG emissions caused by deforestation. The present study conducted an empirical analysis that revealed, for the first time, the impact of the SBP on avoided deforestation by comparing individual and collective SBP contracts. The results indicated that if the SBP had not been implemented, the deforestation rate would have been higher. On the other hand, it is worth mentioning that the results are only related to the effect of the SBP on deforestation. The SBP may have had a broad and positive effect on local ways of life or incomes as well as on the ecological properties of the tropical Andean forest; however, this effect is not quantifiable through categorical variables of the forest. The present study did not attempt to address these issues, but further studies could consider them.

Given the impact of the SBP on society and nature, it is necessary to implement periodic assessment programs to determine not only the role of the SBP on deforestation but also its impact with respect to human wellbeing. In this way, the SBP should be recognized as a national initiative that may contribute to the mitigation of global climate change; therefore, the financing of the SBP for all 20 years of the contracts must be protected through a national and international commitment.

## Supporting information

S1 Table (DOCX)

S1 Fig (DOCX)

S2 Fig (DOCX)
