# Supplementary material for: Are government incentives effective for avoided deforestation in the tropical Andean forest?
Source: PLoS One. 2018 Sep 13;13(9):e0203545. doi: 10.1371/journal.pone.0203545 (PMC6136730; doi:10.1371/journal.pone.0203545)
Supplement: S1 Table — (DOCX) [file pone.0203545.s001.docx]

S1 Table. Sensitivity test of hidden biases measured by critical *p*-values

| Γ | Protected in 2014 (never protected by the SBP and forest in 2008 - control) |
| --- | --- |
| 1.0 | <0.0001 |
| 1.4 | <0.0001 |
| 1.4 | 0.0002 |
| 1.5 | 0.0007 |
| 1.6 | 0.0016 |
| 1.7 | 0.0033 |
| 1.8 | 0.0063 |
| 1.9 | 0.0110 |
| 2.0 | 0.0181 |
| 2.1 | 0.0280 |
| 2.2 | 0.0412 |
| 2.3 | 0.0581 |
| 2.4 | 0.0789 |
| 2.5 | 0.1036 |
| 2.6 | 0.1321 |
| 2.7 | 0.1641 |
| 2.8 | 0.1992 |
| 2.9 | 0.2370 |
| 3.0 | 0.2769 |
| 3.1 | 0.3183 |
| 3.2 | 0.3606 |
| 3.3 | 0.4034 |
| 3.4 | 0.4459 |
| 3.5 | 0.4879 |
| 3.6 | 0.5287 |
| 3.7 | 0.5682 |
| 3.8 | 0.6060 |
| 3.9 | 0.6419 |
| 4.0 | 0.6757 |
