# Supplementary figures and images for: Are government incentives effective for avoided deforestation in the tropical Andean forest?
Source: PLoS One. 2018 Sep 13;13(9):e0203545. doi: 10.1371/journal.pone.0203545 (PMC6136730; doi:10.1371/journal.pone.0203545)

S1 Fig. Boxplot of variables


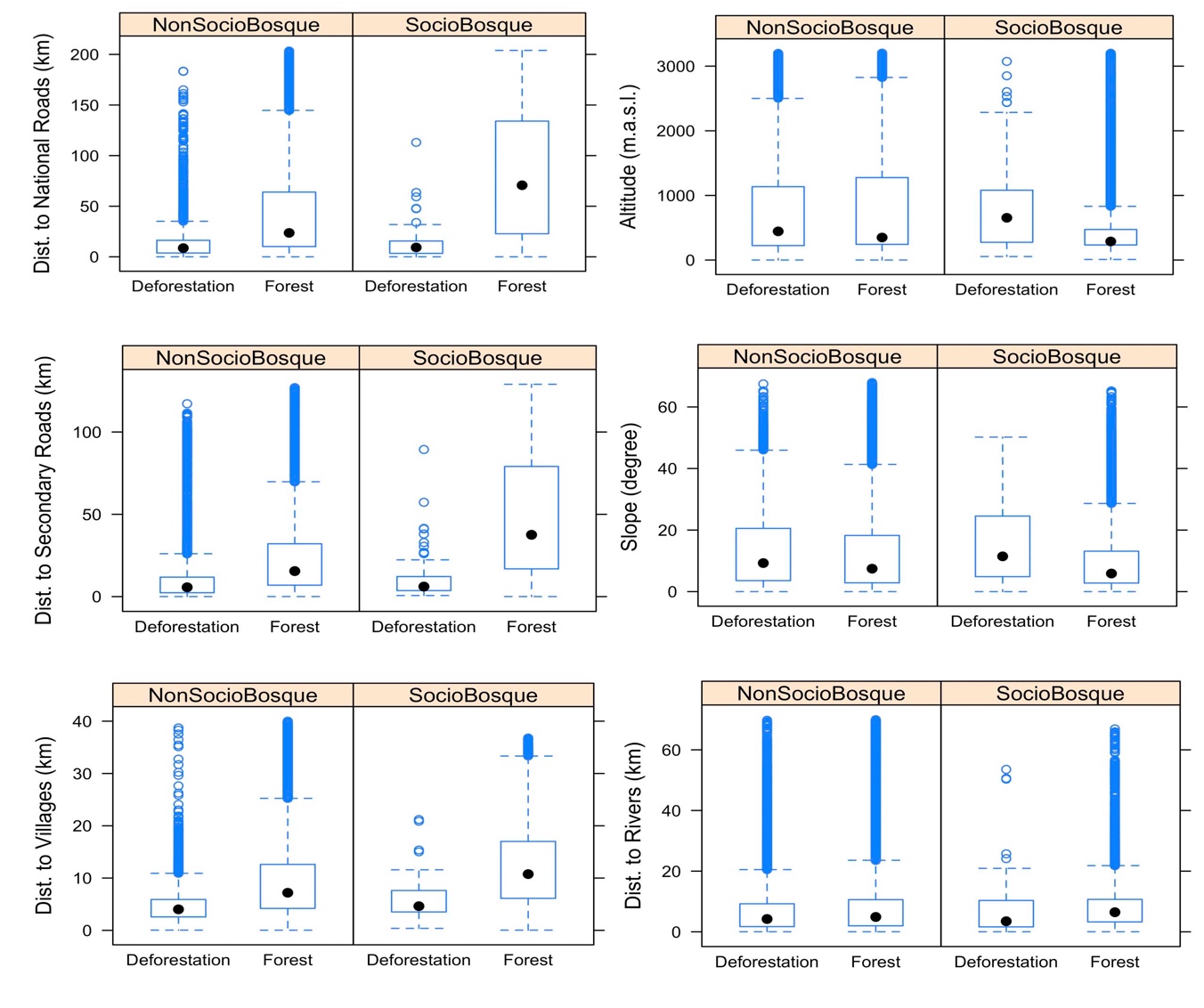

Supplement: S1 Fig — (DOCX) [file pone.0203545.s002.docx]

S2 Fig. Variables before and after matching


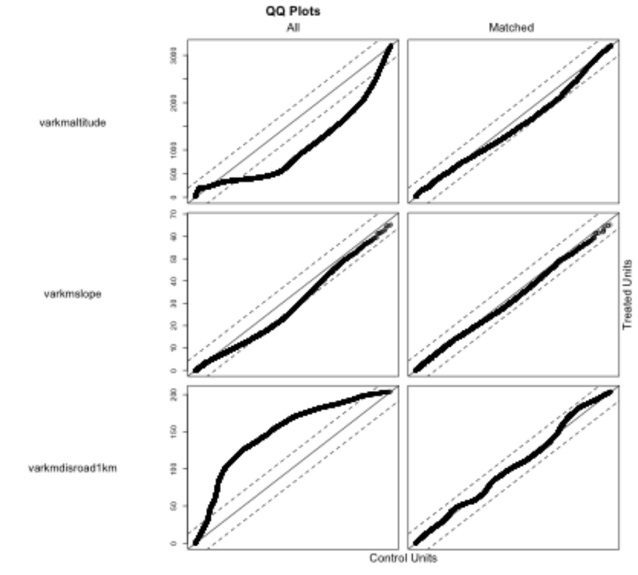


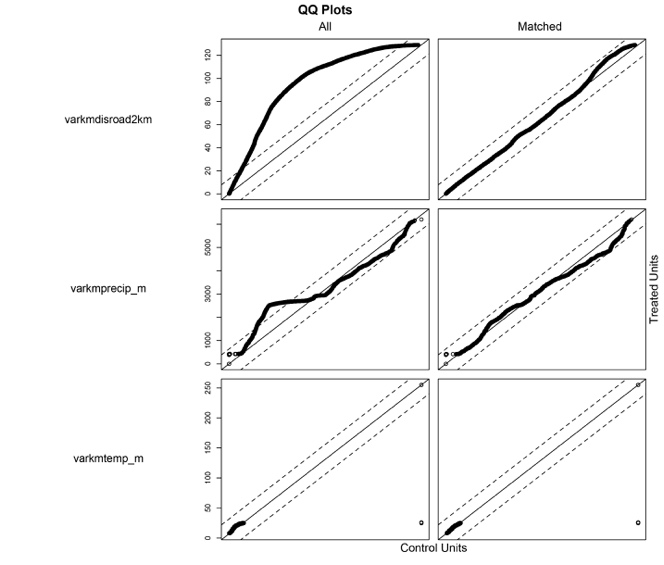


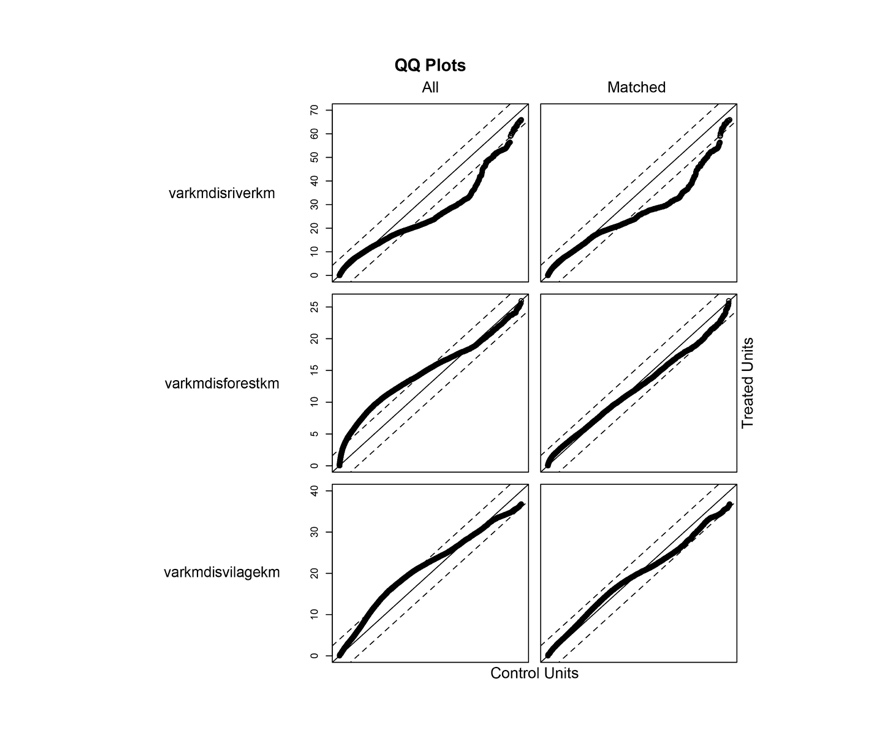

Supplement: S2 Fig — (DOCX) [file pone.0203545.s003.docx]
